# Not only ACE2—the quest for additional host cell mediators of SARS-CoV-2 infection: Neuropilin-1 (NRP1) as a novel SARS-CoV-2 host cell entry mediator implicated in COVID-19

**DOI:** 10.1038/s41392-020-00460-9

**Published:** 2021-01-18

**Authors:** Ioannis Kyrou, Harpal S. Randeva, Demetrios A. Spandidos, Emmanouil Karteris

**Affiliations:** 1 Warwickshire Institute for the Study of Diabetes, Endocrinology and Metabolism (WISDEM), University Hospitals Coventry and Warwickshire NHS Trust, Coventry, CV2 2DX UK; 2 Aston Medical Research Institute, Aston Medical School, College of Health and Life Sciences, Aston University Birmingham, B4 7ET UK; 3 Warwick Medical School, University of Warwick, Coventry, CV4 7AL UK; 4 Laboratory of Clinical Virology, Medical School, University of Crete, 71409 Heraklion, Greece; 5 Biosciences, College of Health, Medicine and Life Sciences, Brunel University London, Uxbridge, UB8 3PH UK

**Keywords:** Infectious diseases, Infectious diseases, Medical research

Two recently published studies published in *Science* by Daly et al.^1^ and Cantuti-Castelvetri et al.^2^ identified neuropilin-1 (NRP1) as an additional cellular mediator which may facilitate the entry of the new severe acute respiratory syndrome (SARS)-coronavirus (CoV)-2 (SARS-CoV-2) into host cells. The findings of these elegant studies collectively indicate that, in addition to the role of angiotensin-converting enzyme 2 (ACE2) in mediating the cellular entry of SARS-CoV-2, NRP1 may act as a host cell mediator that can increase the infectivity and may thus contribute to the tissue/organ tropism of this coronavirus.

SARS-CoV-2 is an enveloped RNA virus that is transmitted mainly via air droplets and causes a viral infectious disease, which was first described at the end of 2019 (i.e., coronavirus disease 2019; COVID-19).^3^ COVID-19 manifestations range from mild (asymptomatic or mild respiratory tract infection in most cases) to severe or even fatal in high-risk individuals, with respiratory and extra-pulmonary manifestations requiring hospitalization and potentially mechanical ventilation and intensive care unit (ICU) support.^3^

**Fig. 1:**
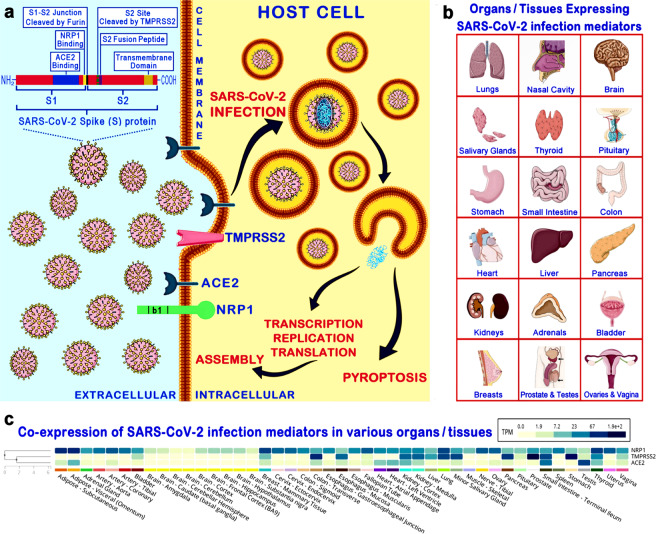
**a** The SARS-CoV-2 spike (S) glycoproteins facilitate the binding and subsequent fusion of this coronavirus with the host cell membrane. These are cleaved (during the host cell entry or the production of SARS-CoV-2 particles) into two components that remain non-covalently linked, namely S1 which constitutes the cell connecting head of this molecule and S2 which remains anchored to the viral membrane and facilitates its fusion. As such, S1 binds to angiotensin-converting enzyme 2 (ACE2) on the cell membrane, whilst S2 mediates the fusion between the viral and host cell membranes after proteolytic exposure of a S2 fusion peptide by transmembrane protease serine 2 (TMPRSS2) or other host cell proteases, such as furin, located within the host cell secretory pathway and endocytic compartments. Indeed, furin cleaves the SARS-CoV-2 S glycoprotein at the S1-S2 junction/boundary which consists of a sequence of basic amino acids. Notably, this furin-mediated cleavage of the polybasic S1-S2 junction exposes a carboxyl-terminal motif [RRAR, where R represents arginine (Arg): Arg-Arg-Ala-Arg], which conforms to the “C-end rule” (CendR) motif [R-XX-R, where R represents arginine (Arg) and can be substituted by lysine (Lys), whilst X can be any amino acid]. This CendR motif of S1 can bind to neuropilin-1 via its b1 domain (NRP1; a pleiotropic single-transmembrane co-receptor for class-3 semaphorins and vascular endothelial growth factors), which is shown to act as a host cell mediator that can increase the SARS-CoV-2 infectivity and contribute to its tissue/organ tropism.^1,2,6^ **b** Expression of ACE2 and other cellular mediators (e.g., TMPRSS2, and NRP1) that facilitate the infection of host cells by SARS-CoV-2 has been noted in various tissues/organs of the human body and may contribute to the tropism of SARS-CoV-2 and corresponding COVID-19 manifestations.^3–5^ **c** Co-expression of ACE2, TMPRSS2, and NRP1 that mediate the SARS-CoV-2 infection of host cells in various human tissues/organs, based on available data from the Genotype-Tissue Expression (GTEx) project

As noted for the closely related SARS-CoV, SARS-CoV-2 entry into human cells is mediated by ACE2, which acts as a cell membrane receptor binding the spike (S1) glycoproteins on the surface of SARS-CoV-2 virions.^4^ In addition, transmembrane protease serine 2 (TMPRSS2) and other proteases (e.g., furin), which are located in the host cell membrane, secretory pathway and endocytic compartments, have also been shown to play a key role by priming the spike proteins of SARS-CoV-2 and facilitating its endocytosis and the release of its viral genome in the infected host cell for subsequent replication (Fig. 1a).^4–6^

Notably, ACE2 exhibits a low/moderate expression in the human respiratory system; thus, research has also focused on identifying additional mediators which may increase SARS-CoV-2 infectivity and may contribute to the tissue/organ tropism of this coronavirus (Fig. 1b, c).^4,5^ Accordingly, data are emerging for a number of cellular mediators/receptors which may also facilitate the host cell infection by SARS-CoV-2, including CD147, glucose-regulated protein 78 (GRP78), angiotensin II receptor type 2 (AGTR2), the receptor for advanced glycation end products (RAGE), heparan sulfate, sialic acids and neuropilin-1 (NRP1).^4,5^

NRPs are highly conserved, non-tyrosine kinase, single-transmembrane glycoproteins, which are expressed in all vertebrates and primarily constitute co-receptors for various molecules (e.g., semaphorins and vascular endothelial growth factors (VEGFs)).^1,2,5,6^ As such, NRPs are involved in diverse physiological processes, including cell proliferation, angiogenesis, vascular permeability, immune functions, neuronal development and axon control.^1,2,5,6^ Of note, peptides/proteins with a carboxyl-terminal sequence, which consists of a conserved motif, known as the “C-end rule” (CendR) [R-XX-R; where R represents arginine (Arg) and can be substituted by lysine (Lys), whilst X can be any amino acid], can bind to and activate NRP1 and NRP2.^1,2,6^

Taking into account these CendR-dependent peptide-NRP interactions, the two recent studies published in Science by Daly et al.^1^ (preprint posted at biorxiv.org in June, 2020; 10.1101/2020.06.05.134114), and Cantuti-Castelvetri et al.^2^ (preprint posted at biorxiv.org in July, 2020; 10.1101/2020.06.07.137802) identified NRP1 as a cellular mediator/receptor which, in addition to ACE2 and TMPRSS2, can facilitate the entry of SARS-CoV-2 into host cells, thus increasing its infectivity and contributing to its tropism. Indeed, these studies indicate that, contrary to the S proteins of SARS-CoV, SARS-CoV-2 S proteins exhibit a polybasic sequence motif (Arg-Arg-Ala-Arg) at the S1-S2 junction/boundary, which conforms to the CendR motif (RRAR) and constitutes a cleavage site for furin (Fig. 1a).^1,2,6^

Accordingly, using human cell lines, these elegant studies demonstrated that, by binding to this CendR motif of the furin-cleaved SARS-CoV-2 S1 protein, NRP1 promotes infection by a clinical SARS-CoV-2 isolate (SARS-CoV-2/human/Liverpool/REMRQ001/2020)^1^ and by lentiviral particles pseudotyped with the S protein of SARS-CoV-2 (pseudoviruses).^2^ This NRP1-mediated enhancement of SARS-CoV-2 infectivity was attributed to increased viral entry into the host cells rather than to higher viral binding to the cell membrane, and was further increased when ACE2 and TMPRSS2 were present.^1,2,6^ Although Daly et al. established that the NRP1 b1 domain/pocket can directly bind a synthetic S1 CendR peptide with micromolar affinity,^1^ the exact molecular mechanisms of the NRP1-mediated effect on SARS-CoV-2 infectivity have not yet been fully elucidated. Nevertheless, it is known that NRPs can facilitate the internalization of CendR peptides/ligands via an endocytic process similar to macropinocytosis.^1^ Notably, NRP1 is also known to play a role in promoting host cell infection by other viruses, such as the Epstein-Barr virus (EBV).^5,6^

Moreover, in these two recent studies, the NRP1-enhanced SARS-CoV-2 infectivity in human cell cultures was inhibited by utilizing a monoclonal blocking antibody against the extracellular NRP1 b1b2 domain or a small-molecule, selective NRP1 antagonist which binds the CendR-binding b1 domain/pocket.^1,2,6^ Similarly, SARS-CoV-2 mutants with an altered furin cleavage site of the S protein (deleted polybasic cleavage site or resistant to furin-mediated cleavage) were not dependent on NRP1 for infectivity, whilst mutations in the NRP1 b1 domain/pocket also inhibited the NRP1-S1 interactions.^1,2,6^

Interestingly, six hours following the intranasal administration of nanoparticles coated with SARS-CoV-2 S-derived CendR peptides to anesthetized adult mice, Cantuti-Castelvetri et al. noted a significant uptake of these, not only in the olfactory epithelium which expresses NRP1, but also into the blood vessels and neurons of the cortex.^2^ The latter further suggests that the broader NRP1 expression in the olfactory epithelium and the human brain, including olfactory-related regions, may also have implications for the manifestations of COVID-19.^5^

In this context, upregulated NRP1gene expression has been also noted in lung tissue of patients with COVID-19.^1^ As such, using antibodies against the SARS-CoV-2 S protein, Cantuti-Castelvetri *et al*. further studied a series of autopsies from six patients with COVID-19 and demonstrated that SARS-CoV-2 infection of the olfactory epithelium was present in five of these cases.^2^ Of note, a high NRP1 expression was noted in the infected olfactory epithelial cells, whilst additional co-staining indicated the infection of cells that were positive for the oligodendrocyte transcription factor 2 (OLIG2; mainly expressed by olfactory neuronal progenitors).^2^

As extra-pulmonary manifestations (e.g., gastrointestinal and neurologic symptoms and complications) are increasingly recognized as part of the COVID-19 pathology,^3,5^ further research is clearly required into the tropism of SARS-CoV-2 in relation to the distribution of its host cell infection mediators across different human tissues/organs (Fig. 1b, c). This is expected to further elucidate the underlying COVID-19 pathophysiology, identify tissues/organs that are more vulnerable to SARS-CoV-2 infection and develop targeted treatments. On this premise, the identification of NRP1 as another cellular mediator which may promote the entry of SARS-CoV-2 into host cells offers a potential novel therapeutic antiviral target against COVID-19.
